# Monomeric Garnet, a far-red fluorescent protein for live-cell STED imaging

**DOI:** 10.1038/srep18006

**Published:** 2015-12-09

**Authors:** Anika Hense, Benedikt Prunsche, Peng Gao, Yuji Ishitsuka, Karin Nienhaus, G. Ulrich Nienhaus

**Affiliations:** 1 Institute of Applied Physics, Karlsruhe Institute of Technology (KIT), 76131 Karlsruhe, Germany; 2 Institute of Nanotechnology, Karlsruhe Institute of Technology (KIT), 76344 Eggenstein-Leopoldshafen, Germany; 3 Institute of Toxicology and Genetics, Karlsruhe Institute of Technology (KIT), 76344 Eggenstein-Leopoldshafen, Germany; 4 Department of Physics, University of Illinois at Urbana-Champaign, Urbana IL 61801, USA

## Abstract

The advancement of far-red emitting variants of the green fluorescent protein (GFP) is crucially important for imaging live cells, tissues and organisms. Despite notable efforts, far-red marker proteins still need further optimization to match the performance of their green counterparts. Here we present mGarnet, a robust monomeric marker protein with far-red fluorescence peaking at 670 nm. Thanks to its large extinction coefficient of 95,000 M^−1^cm^−1^, mGarnet can be efficiently excited with 640-nm light on the red edge of its 598-nm excitation band. A large Stokes shift allows essentially the entire fluorescence emission to be collected even with 640-nm excitation, counterbalancing the lower fluorescence quantum yield of mGarnet, 9.1%, that is typical of far-red FPs. We demonstrate an excellent performance as a live-cell fusion marker in STED microscopy, using 640 nm excitation and 780 nm depletion wavelengths.

In recent years, super-resolution fluorescence microscopy methods have evolved at a fast pace^1^,^2^,^3^,^4^,^5^, and wide-spread application of these techniques is fostered by the availability of commercial equipment. Arguably the most exciting imaging applications involve live specimens, including cells, tissues and entire organisms. Key to success of fluorescence imaging is the availability of bright fluorescent markers that can highlight molecules and structures specifically and efficiently. For live imaging, fluorescent proteins (FPs) of the GFP family play a prominent role because they can be genetically encoded and expressed by the live sample itself as a fusion construct with the protein of interest. This provides a powerful, versatile and simple approach to the specific labeling of live specimens. Great efforts have been made to develop and optimize marker proteins of the GFP family. Many novel hues have been created by genetic engineering, and the color palette has been extended from the blue to the far-red side of the spectrum^6^,^7^,^8^,^9^,^10^. Furthermore, photoactivatable FPs have been developed that are essential for super-resolution localization microscopy (e.g., PALM/STORM)^11^,^12^.

Far-red emitting FPs are advantageous as markers for live specimens for multiple reasons: In biological samples, (1) absorption, (2) scattering and (3) autofluorescence are greatly reduced in the red part of the visible spectrum. These are crucial advantages, especially when investigating samples more complex than single cell layers. Moreover, (4) red light is less phototoxic, and (5) far-red FPs offer the possibility of implementing an additional detection channel for multi-color imaging. Despite the urgent need for far-red FPs, researchers have not yet succeeded in engineering such marker proteins that can compete with the gold standard, EGFP, in their performance in terms of photostability, brightness and extent of maturation. There is still much room for further optimization of far-red FPs^13^.

In this work, we present mGarnet, a robust far-red monomeric FP, which we have found particularly useful for confocal and stimulated emission depletion (STED) imaging applications. In STED microscopy, the image is raster-scanned by a tightly focused excitation beam followed by a donut-shaped depletion beam that causes an effective sharpening of the excitation spot. Any fluorescent marker used for STED must be exquisitely photostable because it undergoes multiple excitation-depletion cycles while the image is scanned near its location. This poses a particular challenge to FPs because they are less photostable than many organic dyes used as fluorescence markers^1^,^14^,^15^. Still, live-cell STED imaging has been demonstrated by using the yellow fluorescent protein, YFP^14^,^16^, and, more recently, the far-red tetrameric E2-Crimson^17^ and monomeric TagRFP657^18^. STED microscopes are typically equipped with 633–640 nm excitation and 750–800 nm emission depletion lasers for excitation of a range of robust dyes that are commercially available. These far-red wavelengths can be utilized with mGarnet as a marker in live imaging experiments. Its properties in terms of brightness, maturation and photostability make mGarnet attractive as a live-cell fluorescence marker in confocal and STED imaging applications. Essential parameters of mGarnet and other FPs with peak fluorescence emission above 600 nm are compiled in Table 1.

## Results

### Protein Engineering

We chose mRuby^19^ as a ‘template’ for the development of a far-red emitting marker protein. mRuby is a bright, monomeric red-fluorescent protein^13^, derived from the tetrameric eqFP611^20^ by extensive protein engineering. Overall, we created more than 300 variants by elaborate rational engineering to finally obtain mGarnet, which differs from mRuby by four point mutations, R67K, T158N, F174L and H197R (sequence numbering according to eqFP611, Supplementary Fig. S1).

In mRuby, the anionic GFP chromophore is in the *trans* isomeric state, and its delocalized π-electron system is extended by an acylimine group to form a red-shifted chromophore with absorption and emission peaks at 558 and 605 nm, respectively (Supplementary Fig. S2a). From the parent protein, eqFP611, it was known that the emission of the chromophore can be shifted to the red by amino acid modifications that stabilize the *cis* with respect to the *trans* configuration^21^. Accordingly, the T158N mutation induced a *trans-cis* isomerization of the chromophore (Supplementary Fig. S2b) and, as in the far-red FP eqFP670^22^, caused a 20-nm red shift of both the absorption and emission maxima^23^.

The general strategy to achieve additional red shifts involves destabilizing the chromophore ground state, stabilizing the excited state, or further extending the conjugated π-electron system. The replacement of phenylalanine by the aliphatic leucine at position 174 was found to facilitate chromophore maturation and to stabilize the red anionic form of the variant mRuby625^23^. In the course of our engineering efforts next to the chromophore, we had realized that H197 is a critical residue. Its replacement by other amino acids has severe effects on protein folding, chromophore maturation, and the emission wavelength. H197 is part of a cluster of four amino acids adjacent to the chromophore which form a planar structure in anthozoan FPs, stabilized by a close-knit network of hydrogen bonding interactions. This rigid scaffold restricts chromophore motions, which are detrimental for the fluorescence quantum yield. In a range of far-red emitting FPs, including mKate^24^,^25^ and its monomeric derivatives mNeptune^26^, mCardinal^27^, and TagRFP675^28^ and also the dimeric eqFP650 and eqFP670^22^, H197 was substituted by an arginine. Consequently, we also introduced this replacement. Unfortunately, this modification had disastrous consequences, leading to a large fraction of misfolded protein as well as incompletely matured chromophores (data not shown). Only after the additional replacement R67K, proper protein folding and chromophore maturation was recovered. Apparently, the smaller lysine side chain at position 67 creates spare volume so that the large R197 side chain can be accommodated (Supplementary Fig. S2b). Together, these two replacements caused further red shifts of the absorption and emission bands to 598 nm and 670 nm, respectively.

### Protein characterization

**Figure 1:**
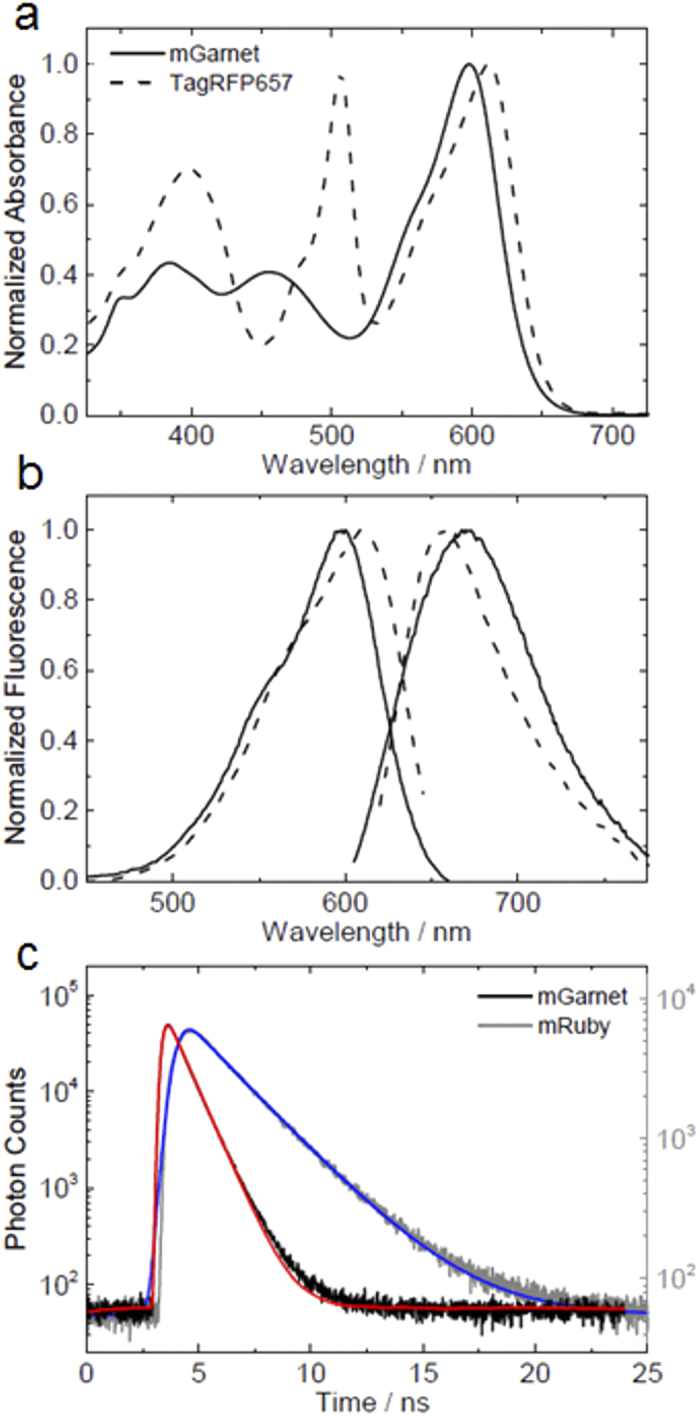
Optical characterization of mGarnet. (**a**) Absorption spectra of mGarnet (solid line) and, for comparison, TagRFP657 (dotted line), (**b**) excitation (λ_em_ = 670 nm) and emission (λ_exc_ = 598 nm) spectra of mGarnet (solid lines) and TagRFP657 (dotted lines). (**c**) Fluorescence intensity decays of mGarnet (black) and mRuby (gray). Solutions of mGarnet and mRuby were excited at 640 nm and 561 nm, respectively, with pulsed lasers running at 40 MHz. Also shown are curves modeling the exponential intensity decay of mGarnet (red) and mRuby (blue), respectively.

The four additional mutations that we introduced into the mRuby sequence did not affect its oligomeric state; mGarnet is a true monomer (Supplementary Fig. S3). The optical spectra shown in Fig. 1 were taken on protein solutions at pH 7.4. Its absorbance spectrum in Fig. 1a shows the strong absorption of the anionic red chromophore at 598 nm, a minor neutral red species at 455 nm, additional absorption from S_0_-S_2_ and, presumably, residual immature species in the near UV. Fluorescence excitation and emission spectra are plotted in Fig. 1b. For comparison, we have also plotted the spectra of TagRFP657, a marker protein that was previously examined for STED imaging^18^. Of note, mGarnet lacks a residual absorption peak near 500 nm that would be indicative of an incompletely matured, anionic green chromophore. Such a population can be a nuisance in dual-color applications together with green FPs. At 37 °C, the maturation half-life of the chromophore, 112 ± 5 min, is in an intermediate range of far-red FPs (Table 1). In proteins of the GFP family, two chromophore species exist, associated with the neutral and anionic forms of the hydroxyphenyl moiety. Often, their populations are controlled by a simple protonation equilibrium. In mGarnet, we determined the chromophore p*K*_*a*_, 7.40 ± 0.05, by measuring the absorption at 598 nm as a function of pH and fitting these data with the Henderson-Hasselbalch equation (Supplementary Fig. S4a). A lower p*K*_*a*_, 6.80 ± 0.05, is obtained on the basis of the pH dependence of the fluorescence emission intensity (Supplementary Fig. S4b). We quote this value here only for comparison with data measured on other FPs in this way, but note that this approach is less reliable because the emission intensity is not only affected by the population of the two species, but also by the intrinsic pH dependence of the fluorescence quantum yield. The anionic *cis* chromophore has an extinction coefficient of 95,000 M^?1^cm^?1^, which is somewhat reduced from the value of 112,000 M^−1^cm^−1^ of mRuby, but still very high for a monomeric red FP (Table 1). The quantum yield of 0.091 ± 0.001 is in the range of other FPs with emission maxima above 640 nm, but significantly lower than for some FPs emitting in the green to yellow range^29^. Presumably, the extended chromophore of far-red FPs is less restrained by the modified protein scaffold. As a consequence, the enhanced dynamics opens non-radiative deexcitation channels, which is also evident from the fluorescence lifetime of mGarnet’s *cis* chromophore of 0.82 ± 0.01 ns (Fig. 1c), which is markedly reduced with respect to that of mRuby (2.6 ± 0.1 ns).

**Figure 2:**
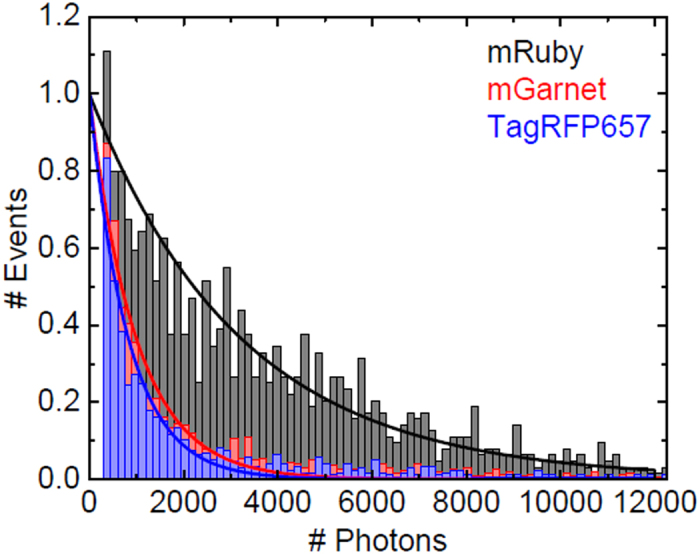
Normalized histogram of FP molecule numbers versus the total number of photons collected from each FP prior to photobleaching. The data were grouped into bins of 150 photon counts. Data below 300 counts were excluded to suppress spurious events and photoswitching to transient dark states. Exponential decay curves (solid lines) yield average photon numbers of 3,198 ± 29, 989 ± 26 and 823 ± 24 for mRuby (black), mGarnet (red) and TagRFP657 (blue), respectively.

A key problem in fluorescence marker applications is the unavoidable photodestruction of the FP chromophore. By using a single-molecule photobleaching assay, we have quantified the persistence of mGarnet against photobleaching in comparison with the red FP mRuby and the far-red TagRFP657. The proteins were immobilized sparsely on PEG surfaces and imaged in total internal reflection mode using a wide-field fluorescence microscope with single-molecule sensitivity^30^ under exactly the same conditions, with excitation by a 561-nm continuous-wave laser. The total number of photons collected from ~2,000 individual molecules per FP prior to photodestruction were plotted in a histogram and fitted with exponential decay functions to yield average photon numbers, 3,189 ± 29 for mRuby, 989 ± 26 for mGarnet and 823 ± 24 for TagRFP657 (Fig. 2). Photobleaching of mGarnet is clearly enhanced with respect to mRuby, but it still emits 20% more photons on average than the far-red TagRFP657 before it photobleaches.

### Super-resolution STED imaging with mGarnet fusion constructs

**Figure 3:**
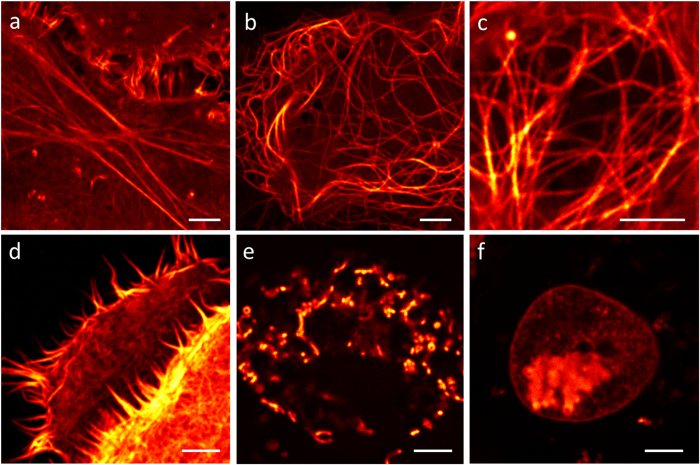
Confocal microscopy images of mGarnet fusion proteins expressed in live COS-7 cells. Cells were transfected with (**a**) LifeAct^31^-mGarnet, (**b**) mGarnet-RITA^32^, (**c**) mGarnet-α-tubulin, (**d**) α-actinin-mGarnet, (**e**) mito-mGarnet and (**f**) H2B-mGarnet. Scale bars, 5 μm.

To probe the performance of mGarnet as a fluorescence marker for live-cell imaging of subcellular structures, we generated six fusion proteins, α-actinin-mGarnet, LifeAct^31^-mGarnet, mGarnet-α-tubulin, mGarnet**-**RBP-J interacting and tubulin associated (RITA) protein^32^, mito-mGarnet and histone-2B-mGarnet (H2B-mGarnet). Figure 3 shows representative confocal images of live COS-7 cells expressing these constructs. Actin and microtubular cytoskeletal structures, mitochondria and nuclei are brightly stained and well resolved under 640-nm excitation. Although we have irradiated into the red wing of the 598-nm absorption band, excitation is very efficient owing to mGarnet’s large extinction coefficient (Table 1). Moreover, a large fraction of the emitted photons can be collected because of its substantial Stokes shift of 72 nm. The confocal images thus attest to the excellent properties of mGarnet as a far-red fusion marker.

**Figure 4:**
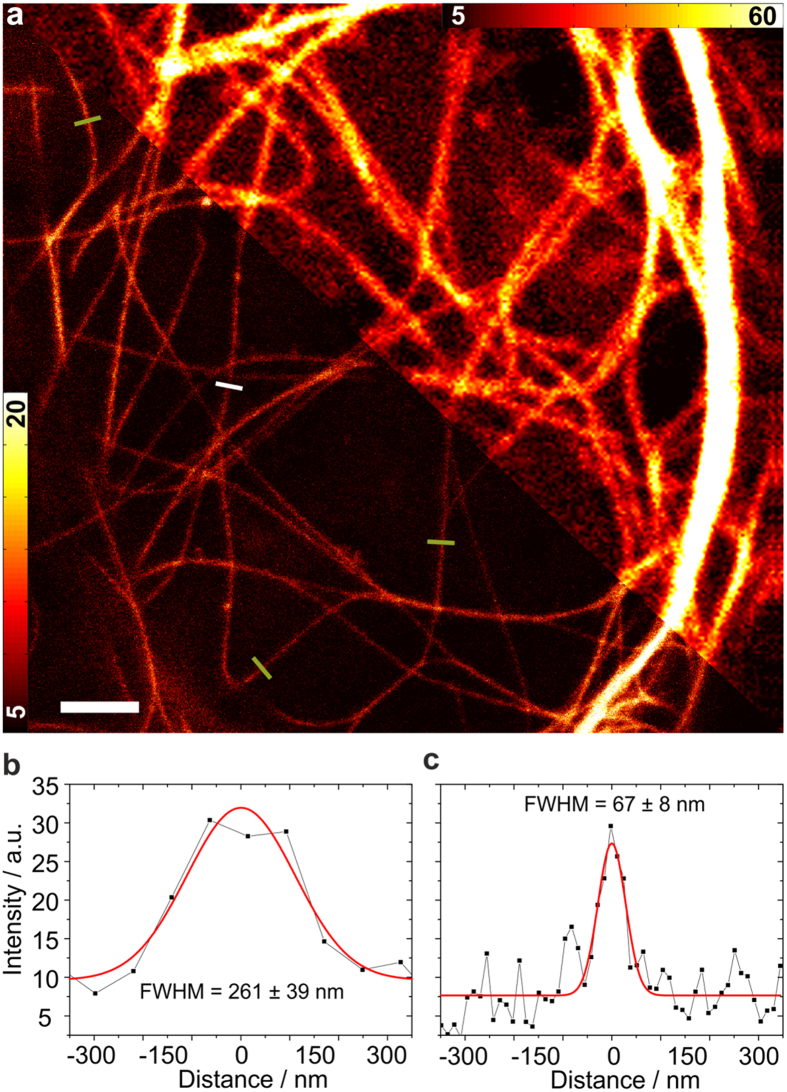
Comparison of confocal and STED imaging using mGarnet-RITA as a marker of microtubules in live COS-7 cells. (**a**) Combined confocal (top) and STED (bottom) image of a cell transfected with the mGarnet-RITA fusion protein (640-nm excitation at 6.1 μW, 780-nm depletion at 56 mW, pixel dwell time 40 μs, pixel size 80 nm (confocal) and 20 nm (STED)). (**b**,**c**) Cross sections of microtubules in (**b**) the confocal and (**c**) the STED image marked by the solid white line. The red lines represent Gaussian fits used to determine the full width at half-maximum (FWHM) values. Cross-sections marked by the yellow lines also yielded FWHM values of 60?70 nm (in the STED image). Color bars, counts per pixel. Scale bar, 2 μm.

Next, we imaged microtubular structures stained with mGarnet**-**RITA in standard confocal and STED mode (Fig. 4a). For STED imaging, we used a Ti:Sa laser operated at 780 nm and 56 mW for emission depletion in addition to the 640-nm pulsed diode laser (power 6.1 μW) for excitation. A substantial resolution improvement with STED microscopy is visible (Fig. 4a, top/bottom). For quantification of the sharpness of the features, we calculated several cross-sections of microtubules, as indicated by the white lines in Fig. 4a, which we averaged over the short extension (not drawn to scale) and fitted with Gaussian distributions to determine the full width at half maximum (FWHM) values. Representative cross-sections (corresponding to the solid line in Fig. 4a) are depicted in Fig. 4b,c. A microtubule is a well-defined filamentous structure with a diameter of ~25 nm^33^, which appears in the cross-section with a FWHM of ~260 nm in the confocal image and ~67 nm in the STED image. Given the size of the microtubule plus its increase due to the additional decoration with mGarnet**-**RITA, we can safely state that an almost 6-fold resolution enhancement was achieved. Comparisons of confocal and STED images of COS-7 cells expressing α-actinin, H2B and LifeAct fused to mGarnet are shown in Supplementary Figs S5–7. In all cases, the STED images displayed a markedly enhanced resolution of the imaged structures. For example, in the images of labeled actin (Supplementary Fig. S7), two neighboring filaments separated by only 181 nm are well resolved in the STED image. We note that all STED images were acquired with a pixel size of 15–20 nm, so that the mGarnet proteins were exposed to a large number of excitation-depletion cycles. To assess the usefulness of mGarnet in live-cell STED imaging of cytoskeletal dynamics over extended periods of time, we further recorded a series of ten images of a COS-7 cell expressing LifeAct-mGarnet in succession, with time intervals of 3 min between successive images (Supplementary Fig. S8). Movements of the actin cytoskeleton are evident, and there is no obvious deterioration of the image quality with time.

### Extended light exposure of mGarnet-labeled cells

**Figure 5:**
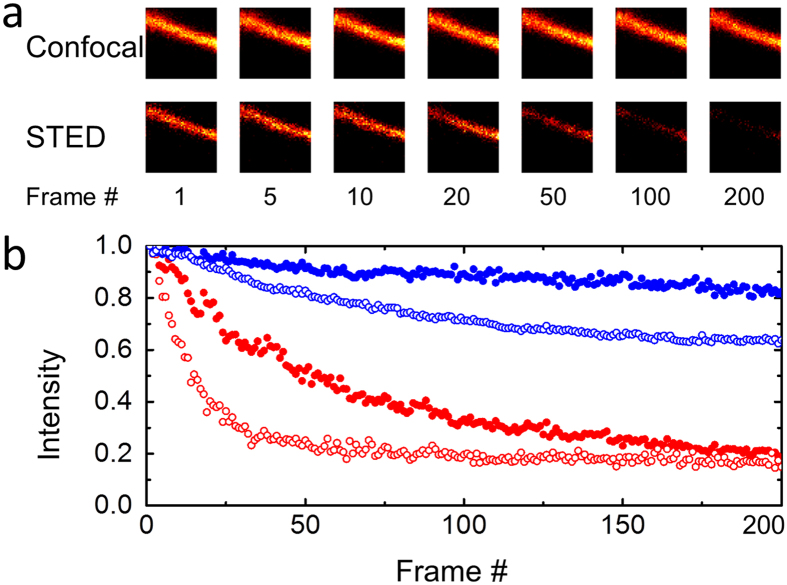
Photobleaching effects studied by repeated confocal and STED imaging of live COS-7 cells labeled with Lifeact-mGarnet. (**a**) From a sequence of 200 successive confocal and STED images, selected images (2 × 2 μm^2^, pixel dwell time 40 μs, pixel size 40 nm) taken with confocal (excitation at 640 nm, laser power 6.1 μW) and STED illumination (excitation at 640 nm, laser power 6.1 μW; depletion at 780 nm, laser power 28.8 mW) are shown. (**b**) Integrated emission intensities, normalized to 1 at the first data point and plotted as a function of image number (blue: confocal, red: STED). Closed symbols: laser irradiation as quoted for panel (**a**); Open blue symbols: laser power increased to 27 μW. Open red symbols: STED depletion laser power increased to 56 mW.

As a robust, quantitative test of the performance of mGarnet in long-term imaging experiments, we collected several sets of 200 confocal and 200 STED images of individual filopodia of a live COS-7 cell expressing LifeAct-mGarnet in succession. These thin protrusions are advantageous for quantitation because they can be imaged essentially without any background. As filopodia are highly dynamic (Supplementary Fig. S8), the overall measurement time needs to be brief. Therefore, we chose an image size to 2 × 2 μm^2^ (a representative region is marked by the white frame in Supplementary Fig. S8), so that it takes only 20 s to collect 200 images (pixel dwell time 40 μs, pixel size 40 nm). Figure 5 shows seven confocal and seven STED images out of a time series. The emission intensities within each image were integrated, corrected for background, and normalized to the intensity of the first image. These data are shown in Fig. 5 as a function of the image number. For confocal imaging under high power, the intensity decreased to ~60% after 200 exposures (Fig. 5, blue symbols). With the depletion beam switched on, the intensity in the images decayed more quickly (Fig. 5, red symbols). Notably, after ~100 STED images at high depletion laser power, the intensity remained stationary at ~20% of the initial value, suggesting that photobleaching is counterbalanced by exchange of bleached LifeAct-mGarnet fusion proteins by new ones on the actin fibers in the live cell.

We also collected a sequence of 200 confocal and STED images in a larger region (20 × 20 μm^2^) of a live COS-7 cell expressing mGarnet-RITA. With a pixel dwell time of 40 μs and a pixel size of 40 nm, it took ~10 s to acquire a single image. The microtubules were less dynamics than the actin structures visualized in Fig. 5, allowing much longer data acquisition times. Supplementary Fig. 9a,b shows confocal images acquired during the first and the 40^th^ scan. The labeled structures appear essentially identical, just less intense. After 200 scans, the integrated emission intensity had decreased to ~50% of its initial value (Supplementary Fig. 9e). With the depletion beam switched on, the intensity in the STED images decayed more rapidly, *i.e.*, to 50% already after 40 scans (Supplementary Fig. 9e). Nevertheless, the intensity was still sufficient for imaging with a resolution of 70–90 nm (Supplementary Fig. 9c,d).

## Discussion

FP markers that can be excited with light wavelengths above 600 nm offer significant advantages, especially for live-cell imaging of thick samples such as tissues and entire organisms, where they provide greater penetration depth and reduced scattering. Here we have shown that our new far-red FP, mGarnet, has attractive properties as a fusion marker in such applications. Even though its excitation band peaks at 598 nm, it can still be excited efficiently with 640-nm light because of its comparatively high peak extinction coefficient (Table 1). Its enormous Stokes shift of 72 nm results in an emission peak at 670 nm, so that, even with red-edge excitation at 640 nm, practically the entire emission band can be admitted into the detector. This compensates for the lower quantum yield of 9.1%, which is, however, typical of current far-red FPs. In combination, the beneficial excitation and emission properties ensure that mGarnet can be employed as a bright fluorescence marker. Of note, mGarnet does not show an incompletely matured, green fluorescent population that is often visible in red FPs, making mGarnet well suited for multi-color imaging. The color shifting modifications led to a lower photostability of mGarnet with respect to its parent protein, mRuby. However, it performed very well in comparison with another far-red FP. By using a variety of fusion marker constructs, we demonstrated an excellent performance of mGarnet as a fluorescence marker for confocal microscopy and STED. Notably, we observed a robust behavior of mGarnet in standard confocal as well as STED imaging applications using popular far-red imaging settings with the widely employed 640 nm excitation >750 nm depletion. In conclusion, although the physical properties of mGarnet still leave room for improvement, this bright far-red FP constitutes a significant advance and appears very useful as a fusion marker for far-red confocal/STED microscopy.

## Methods

### Mutagenesis and fusion gene construction

Point mutations were introduced by using the QuickChange II Site-Directed Mutagenesis Kit (Stratagene, Heidelberg, Germany). The pQE32 vector (Qiagen, Hilden, Germany) with the mRuby cDNA cloned into the *BamHI-XbaI* sites was used as a template. Primers were ordered from Biomers (Ulm, Germany).

To generate mGarnet fusion constructs, the codon-optimized cDNA for mammalian expression of mGarnet was PCR amplified using primers containing the appropriate restriction enzyme sites. For N-terminal fusions, the mGarnet PCR products and the pcDNA3.1 vectors containing the gene sequence of the fusion partners were digested with *XhoI* and *XbaI* (human histone 2B (H2B)), *PvuI* and *NotI* (human α-actinin), *EcoRI* and *XbaI* (human cytochrome c oxidase subunit VIII, *i.e.*, the mitochondrial targeting sequence mito) and *XhoI* and *XbaI* (LifeAct (F-actin marker)). RBP-J Interacting and tubulin-associated (RITA) protein and α-tubulin were fused to the C-terminal end of mGarnet via *KpnI* and *EcoRI* and *NheI* and *XbaI,* respectively. Ligation was performed using the Quick Ligation Kit (NEB, Frankfurt am Main, Germany). Ligation products were transformed and amplified in *E. coli* XL1 and purified using the Pure Yield^TM^ Plasmid Miniprep System Kit (Promega, Mannheim, Germany). DNA Sequencing was carried out by GATC Biotech AG (Konstanz, Germany).

### Protein purification and characterization

The fluorescent proteins were expressed in *E. coli* XL1, purified using a Co^2+^ metal affinity resin column (Clontech, Saint-Germain-en-Laye, France) and desalted using a PD-10 desalting column (GE Healthcare Life Sciences, Munich, Germany)^20^,^34^. For native PAGE, 5 μg of purified proteins were mixed with 3 × loading buffer (50 mM Bis-Tris, 45% (v/v) Glycerin, pH 7.0) and loaded onto a 4?20% Tris polyacrylamide gel (Bio-Rad) and electrophoresed in anode buffer (50 mM Bis-Tris, pH 7.0) and cathode buffer (15 mM Bis-Tris, 5 mM Tricin, 0.02%w/v) Serva-Blue G250, pH 7.0).

Absorbance spectra were measured on a Cary 100 UV/vis spectrophotometer (Varian, Darmstadt, Germany); extinction coefficients were calculated using the base-denaturation method^35^. pH titrations were performed by dissolving the FP in the appropriate buffer solution (40 mM C_6_H_8_O_7_/NaC_6_H_7_O_7_, 300 mM NaCl for pH 3.0?6.0; 40 mM NaH_2_PO_4_/Na_2_HPO_4_, 300 mM NaCl for pH 6.2?8.7; 40 mM NaHCO_3_/Na_2_CO_3_, 300 mM NaCl for pH 8.8?11.0). Excitation and emission spectra were measured with a Horiba Jobin Yvon Fluorolog 3 spectrofluorometer (HORIBA Scientific, Unterhaching, Germany). Quantum yields were determined using eqFP670 as a reference (reported quantum yield 0.06^22^).

To determine the chromophore maturation half-life, *E. coli* M15 cells transformed with the pQE32 vector coding for the FP were grown overnight at 37 °C in DYT medium containing ampicillin (75 mg/L) and kanamycin (25 mg/L) (both from Carl Roth, Karlsruhe, Germany). The cell suspensions were diluted to an optical density of 1.0 at 600 nm, and protein expression (for 1 h at 37 °C) was induced by 1 mM isopropyl β-D-1-thiogalactopyranoside (IPTG, Carl Roth). To restrict the oxygen supply, the cell suspension was sealed in 50 mL tubes filled to the brim. The protein was purified as described above within 40 min at temperatures not exceeding 4 °C. Chromophore maturation at 37 °C was tracked by measuring the emission intensity at 670 nm as a function of time.

Fluorescence lifetimes were measured by time-correlated single-photon counting on a confocal microscope (Microtime 200, PicoQuant, Berlin, Germany). A pulsed 640 nm diode laser (LDH-P-C-640B, PicoQuant) delivering 8 μW at 40 MHz was used for excitation of the protein solution. The emitted light, limited to wavelengths >655 nm by a long pass filter, was focused onto an avalanche photodiode (SPCM-AQR-13, Perkin Elmer, Rodgau, Germany). Data acquisition was controlled by commercial software (SymPhoTime, PicoQuant). Fluorescence intensity decay curves were compiled and subsequently fitted with a routine written in MATLAB R2014 (The Mathworks, Natick, NY, USA), using a convolution of a mono-exponential decay with the instrumental response function.

### Single molecule photobleaching experiments

To determine the average number of photons emitted by individual FP molecules prior to photobleaching, FP molecules were sparsely immobilized via neutravidin/biotin coupling onto polymer-coated quartz coverslips (30:1 mPEG-SVAl:biotin-PEG_SC (w/w), MW 5000, Laysan Bio Inc., Arab, USA) and imaged by an objective-based total internal reflection fluorescence inverted microscope (Axiovert 200, Zeiss, Germany)^30^. For protein biotinylation, 25 μM FP and 250 μM NHS-PEG4-biotin (EZ-Link^TM^ NHS-PEG4-biotin, Life Technologies, Darmstadt, Germany) were mixed in PBS. NaHCO_3_ was added to the final concentration of 100 mM to adjust the pH to 7.4. After gently shaking the solution at room temperature for 2 h, free PEG4-biotin molecules were removed using a desalting column (NAP5, GE Healthcare Life Sciences or Micro Bio-Spin 6, Bio-Rad, Munich, Germany). The biotin-PEG/PEG-coated coverslip was incubated with 100 nM Neutravidin (Pierce^TM^ Neutravidin, Life Technologies) solution for 5 min and washed with PBS. Biotinylated FPs (40?100 pM in PBS) were immobilized on the surface by incubating with the FP solution for 15 min. Excess FP molecules were flushed away with PBS. The sample was illuminated by a 561-nm laser (GCL-150-561, 272 W/cm^2^, CrystaLaser, Reno, NV, USA) in total internal reflection mode (imaged area 256 × 256 pixels, 28.2 × 28.2 μm^2^). The emitted photons were collected with an oil immersion objective (alpha Plan-Apochromat 63x/1.46 Oil Corr M27, Zeiss), passed through a 561-nm longpass filter (EdgeBasic, AHF, Tübingen, Germany) and detected by an EMCCD camera (iXon Ultra 897, Andor Technology Ltd., Belfast, UK) with an exposure time of 50 ms/frame. Laser excitation was synchronized with image acquisition. Data were analyzed using custom written analysis software running under the MATLAB R2014 (The Mathworks, Natick) environment. Single FPs in the raw image frames were identified by using a-livePALM^36^, a custom software written to analyze super-resolution microscopy data. Molecules identified in different frames were linked by applying proximity thresholding (<100 nm). Detected events above a threshold of 300 photons were used to build histograms of the observed numbers of emitted photons from the FP molecules. The histograms were fitted with an exponential decay function.

### Mammalian cell culture and transfection

COS-7 cells (Sigma-Aldrich, St. Louis, USA) were cultured in Dulbecco’s Modified Eagle’s Medium (DMEM) supplemented with 10% fetal bovine serum (FBS) and antibiotics (60 μg/mL penicillin and 100 ng/mL streptomycin, both from Invitrogen, Carlsbad, Canada) at 37 °C and 5% CO_2_. 24 h after seeding the cells on an 8-well Lab-Tek II chambered cover glass (Thermo Fischer Scientific, Waltham, USA), the cells were transfected with 3 μg of DNA by using Lipofectamine® (Invitrogen). The cells were incubated for at least 24 h before imaging.

### STED microscopy of mammalian cells

Living COS-7 cells transfected with mGarnet fusion constructs were imaged in DMEM with 10% FBS without antibiotics, using a home-built STED microscope^37^. Excitation light was generated by a 640-nm pulsed diode laser (LDH-P-C-640B; PicoQuant), emitting 100-ps pulses at 80 MHz. The excitation light was spectrally filtered by a 640/14 nm bandpass and spatially cleaned by 2 m of single-mode polarization-maintaining fiber (PMJ-A3HPC, OZ Optics, Ottawa, Canada). The depletion light was delivered by an 80-MHz mode-locked Ti:Sa laser (Mai Tai HP; Newport Spectra-Physics, Darmstadt, Germany) tuned to 780 nm. The 100-fs pulses of the Ti:Sa laser were stretched by 60 cm of SF6 glass and 100 m of polarization maintaining single mode fiber (PMJ-A3HPC, OZ Optics, Ottawa, Canada) to 300 ps. A vortex phase pattern was loaded onto a spatial light modulator (PLUTO Phase Only, HoloEye, HOLOEYE Photonics AG, Berlin-Adlershof, Germany) generating the doughnut shape of the depletion beam in the focal plane. Excitation and depletion beams were passed through a beam scanner (Yanus IV, Till Photonics, Germany) and a quarter-wave plate, and focused by a microscopic objective (HCX PL APO CS X100/1.46; Leica, Wetzlar, Germany) onto the sample. Fluorescence light was collected by the same objective, separated by a quad-band dichroic mirror (zt405/488/561/640rpc; Chroma Technology Corp, Bellows Falls, USA) and focused into a multimode fiber serving as a confocal pinhole, with the core diameter corresponding to one Airy unit. Subsequently, the fluorescence was filtered by a 700-nm shortpass filter (ET700SP; Chroma Technology Corp) followed by a 676/37 nm bandpass filter (HC 676/37, Semrock, Rochester, NY, USA) to block scattered excitation and STED photons. Photons were detected by an avalanche photodiode (tau-SPAD-50; PicoQuant) and registered by a data acquisition card (PCI-6259; National Instruments, Munich, Germany). STED images were raster-scanned with a pixel dwell time of 40 μs and, unless noted otherwise, a pixel size of 20 nm. Confocal images were taken with identical settings right after acquisition of the STED images.

## Additional Information

**How to cite this article**: Hense, A. *et al.* Monomeric Garnet, a far-red fluorescent protein for live-cell STED imaging. *Sci. Rep.* **5**, 18006; doi: 10.1038/srep18006 (2015).

## Supplementary Material

Supplementary Information

## Figures and Tables

**Table 1:** Properties of red FPs with emission maxima above 600 nm.

Protein	Ex/Em (nm)	QY	EC (M^−1^cm^−1^)	MB relative to mRuby (%)	pK*a*	Maturation half-time at 37 °C (min)	Fluorescence Lifetime (ns)
Monomers							
mRuby	558/605	(0.35)^19^	(112,000)^19^	100	(4.4)^19^	(168)^19^	(2.6)^19^
mRaspberry	598/625	(0.15)^25^	(79,000)^25^	30	(5.0)^17^	(126)^17^	ND
mKate2	588/633	(0.40)^38^	(62,500)^38^	64	(6.5)^17^	(48)^17^	ND
mKate	588/635	(0.28)^38^	(31,500)^38^	23	(6.5)^25^	(75)^25^	ND
mPlum	590/649	(0.10)^25^	(22,000)^25^	6	(5.5)^17^	(96)^17^	ND
mNeptune1	600/650	(0.20)^26^	(67,000)^26^	34	(5.4)^26^	(28)^27^	ND
mNeptune2	599/651	(0.24)^27^	(89,000)^27^	54	(6.3)^27^	(27)^27^	ND
mNeptune2.5	599/643	(0.28)^27^	(95,000)^27^	68	(5.8)^27^	(26)^27^	ND
mCardinal	604/659	(0.19)^27^	(87,000)^27^	42	(5.3)^27^	(27)^27^	ND
TagRFP657	611/657	(0.10)^18^	(34,000)^18^	9	(5.0)^18^	(125)^18^	1.4
mGarnet	598/670	0.09	95,000	22	7.4	112	0.8
Dimers							
eqFP650	592/650	(0.24)^22^	(65,000)^22^	40	(5.7)^22^	ND	ND
eqFP670	605/670	(0.06)^22^	(70,000)^22^	11	(4.5)^22^	ND	ND
Tetramers							
eqFP611	559/611	(0.45)^20^	(78,000)^20^	90	ND	ND	(2.5)^20^
RFP639	588/639	(0.18)^35^	(69,000)^35^	32	(<4.0)^35^	(90)^35^	ND
E2-Crimson	605/646	(0.12)^17^	(58,500)^17^	18	(4.5)^17^	(26)^17^	ND

Molecular Brightness (MB) is a product of quantum yield (QY) and extinction coefficient (EC). ND, not determined.
