# Supplementary material for: Monomeric Garnet, a far-red fluorescent protein for live-cell STED imaging
Source: Sci Rep. 2015 Dec 9;5:18006. doi: 10.1038/srep18006 (PMC4673609; doi:10.1038/srep18006)
Supplement: Supplementary Information [file srep18006-s1.docx]

**Monomeric Garnet, a far-red fluorescent protein for live-cell STED imaging**

Anika Hense, Benedikt Prunsche, Peng Gao, Yuji Ishitsuka, Karin Nienhaus, G. Ulrich Nienhaus

**Supplementary figures:**

| **Supplementary Figure S1** | Sequence alignment of selected RFPs (sequence numbering according to eqFP611). |
| --- | --- |
| **Supplementary Figure S2** | Structural depictions of the fluorophore in mRuby and mGarnet. |
| **Supplementary Figure S3** | Polyacrylamide gel run under native conditions to determine the mGarnet oligomerization state. |
| **Supplementary Figure S4** | pH dependence of the mGarnet chromophore. |
| **Supplementary Figure S5** | Fluorescence images of live COS-7 cells transfected with α-actinin**-**mGarnet. |
| **Supplementary Figure S6** | Fluorescence images of a cell nucleus labeled with H2B-mGarnet. |
| **Supplementary Figure S7** | Confocal and STED images of actin cytoskeletal structures. |
| **Supplementary Figure S8** | Series of STED images of a live COS-7 cell transfected with LifeAct-mGarnet. |
| **Supplementary Figure S9** | Repeated confocal and STED measurements on COS-7 cells labeled with mGarnet-RITA. |

10 20 30 40 50 60

| | | | | |

eqFP611 MNSLIKENMRMMVVMEGSVNGYQFKCTGEGDGNPYMGTQTMRIKVVEGGPLPFAFDILAT

RFP639 MNSLIKENMRMMVVMEGSVNGYQFKCTGEGDGNPYMGTQTMRIKVVEGGPLPFAFDILAT

mRuby MNSLIKENMRMKVVLEGSVNGHQFKCTGEGEGNPYMGTQTMRIKVIEGGPLPFAFDILAT

**mGarnet** MNSLIKENMRMKVVLEGSVNGHQFKCTGEGEGNPYMGTQTMRIKVIEGGPLPFAFDILAT

mKate MSELIKENMHMKLYMEGTVNNHHFKCTSEGEGKPYEGTQTMRIKVVEGGPLPFAFDILAT

eqFP650 MGEDSELISENMHMKLYMEGTVNGHHFKCTSEGEGKPYEGTQTAKIKVVEGGPLPFAFDILAT

eqFP670 MGEDSELISENMHTKLYMEGTVNGHHFKCTSEGEGKPYEGTQTCKIKVVEGGPLPFAFDILAT

TagRFP657 MSELITENMHMKLYMEGTVNNHHFKCTSEGEGKPYEGTQTQRIKVVEGGPLPFAFDILAT

mCardinal MVSKGEELIKENMHMKLYMEGTVNNHHFKCTTEGEGKPYEGTQTQRIKVVEGGPLPFAFDILAT

70 80 90 100 110 120

| | | | | |

eqFP611 SFMYGSKTFIKHTKGIPDFFKQSFPEGFTWERVTRYEDGGVFTVMQDTSLEDGCLVYHAK

RFP639 SFMYGSKTFIKHTKGIPDFFKQSFPEGFTWERVTRYEDGGVFTVMQDTSLEDGCLVYHAK

mRuby SFMYGSRTFIKYPKGIPDFFKQSFPEGFTWERVTRYEDGGVITVMQDTSLEDGCLVYHAQ

**mGarnet** SFMYGSKTFIKYPKGIPDFFKQSFPEGFTWERVTRYEDGGVITVMQDTSLEDGCLVYHAQ

mKate SFMYGSKTFINHTQGIPDFFKQSFPEGFTWERVTTYEDGGVLTATQDTSLQDGCLIYNVK

eqFP650 SFMYGSKTFINHTQGIPDFFKQSFPEGFTWERITTYEDGGVLTATQDTSLQNGCLIYNVK

eqFP670 SFMYGSKTFINHTQGIPDFFKQSFPEGFTWERITTYEDGGVLTATQDTSLQNGCLIYNVK

TagRFP657 SFMYGSHTFINHTQGIPDFWKQSFPEGFTWERVTTYEDGGVLTATQDTSLQDGCLIYNVK

mCardinal CFMYGSKTFINHTQGIPDFFKQSFPEGFTWERVTTYEDGGVLTVTQDTSLQDGCLIYNVK

130 140 150 160 170 180

| | | | | |

eqFP611 VTGVNFPSNGAVMQKKTKGWEPNTEMLYPADGGLRGYSQMALNVDGGGYLSCSFETTYRS

RFP639 VTGVNFPSNGAVMQKKTKGWEPSTEMLYPADGGLRGYCQMALNVDGGGYLFCSFETTYRS

mRuby VRGVNFPSNGAVMQKKTKGWEPNTEMMYPADGGLRGYTHMALKVDGGGHLSCSFVTTYRS

**mGarnet** VRGVNFPSNGAVMQKKTKGWEPNTEMMYPADGGLRGYNHMALKVDGGGHLSCSLVTTYRS

mKate IRGVNFPSNGPVMQKKTLGWEASTEMLYPADGGLEGRSDMALKLVGGGHLICNLKTTYRS

eqFP650 INGVNFPSNGPVMQKKTLGWEASTEMLYPADSGLRGHSQMALKLVGGGYLHCSLKTTYRS

eqFP670 INGVNFPSNGPVMQKKTLGWEANTEMLYPADSGLRGHNQMALKLVGGGYLHCSLKTTYRS

TagRFP657 IRGVNFPSNGPVMQKKTLGWEAHTEMLYPADGGLEGRTALALKLVGGGHLICNFKTTYRS

mCardinal LRGVNFPSNGPVMQKKTLGWEATTETLYPADGGLEGRCDMALKLVGGGHLHCNLKTTYRS

190 200 210 220 230

| | | | |

eqFP611 KKTVENFKMPGFHFVDHRLERLEESDKEMFVVQHEHAVAKFCDLPSKLGRL

RFP639 KKTDENFKMPGFHFVDHRLERLEESDKEMFVVQHEHAVAKFCDLPSKLGRL

mRuby KKTVGNIKMPGIHAVDHRLERLEESDNEMFVVQREHAVAKFAGLGGG

**mGarnet** KKTVGNIKMPGIHAVDRRLERLEESDNEMFVVQREHAVAKFAGLGGG

mKate KKPAKNLKMPGVYYVDRRLERIKEADKETYVEQHEVAVARYCDLPSKLGHKLN

eqFP650 KKPAKNLKMPGFYFVDRKLERIKEADKETYVEQHEMAVARYCDLPSKLGHS

eqFP670 KKPAKNLKMPGFYFVDRKLERIKEADKETYVEQHEMAVARYCDLPSKLGHS

TagRFP657 KKPAKNLKMPGVYYVDYRLERIKEADKETYVEQHEVAVARYCDLPSKLGHKLN

mCardinal KKPAKNLKMPGVYFVDRRLERIKEADNETYVEQHEVAVARYCDLPSKLGHKLNGMD

**Supplementary Fig. S1**. **Sequence alignment of selected RFPs (sequence numbering according to eqFP611).** Conserved residues are shaded in gray. The chromophore tripeptide is marked in purple. The four residues 67, 158, 174 and 197 modified in mRuby to obtain mGarnet are highlighted in turquoise.


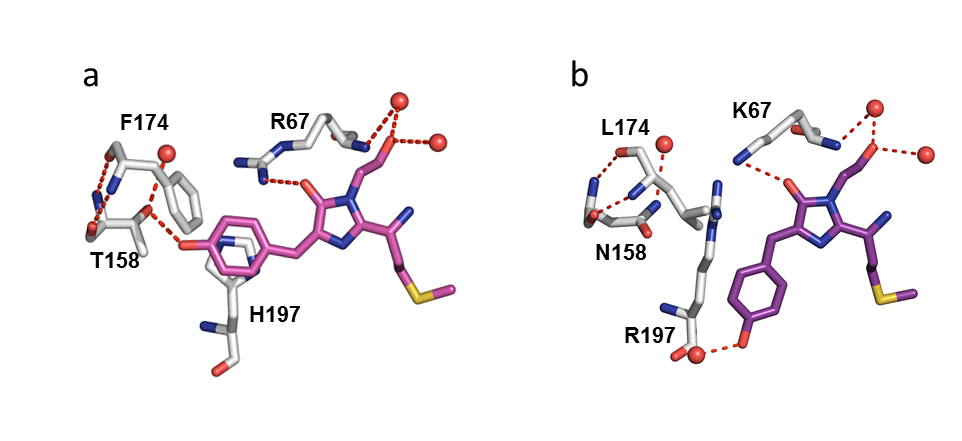


**Supplementary Fig. S2.** **Structural depictions of the chromophores and their environment in mRuby and mGarnet.** (a) In mRuby (pH 8.5), the chromophore adopts the *trans* configuration (magenta) and forms hydrogen bonds (dashed lines) to R67, T158 and two water molecules (PDB accession code 3U0M)[^1^](#_ENREF_1). (b) In mGarnet, as in mRuby (pH 4.5, PDB 3U0L)[^1^](#_ENREF_1), the chromophore presumably adopts the *cis* configuration (purple), in which the neutral hydroxyphenyl moiety is stabilized by a hydrogen bond to a water molecule The modified K67, N158, L174 and R197 side chains were introduced into the structure of mRuby by using the PYMOL mutagenesis tool.


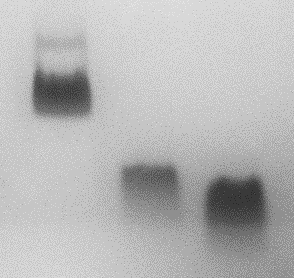


eqFP670

mRuby

mGarnet

dimer

monomer

**Supplementary Fig. S3**. **Polyacrylamide gel run under native conditions to determine the oligomerization state of mGarnet.** The dimeric eqFP670[^2^](#_ENREF_2) and the monomeric mRuby were used as references.


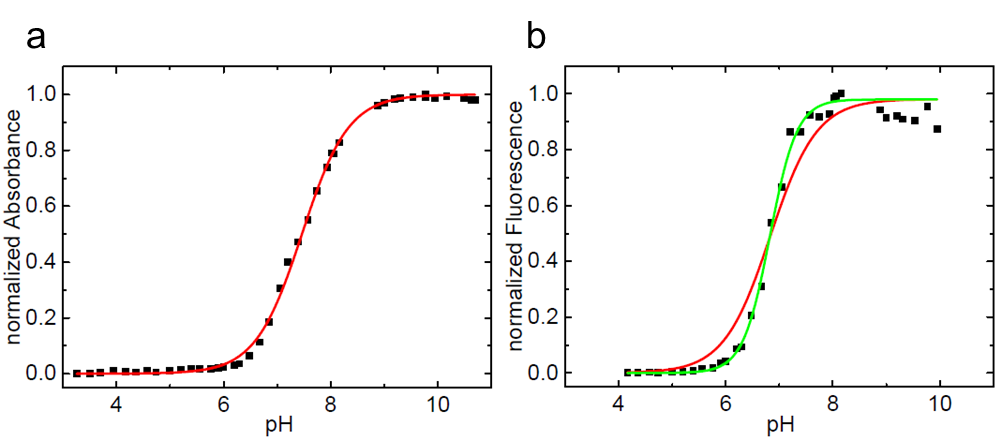


**Supplementary Fig. S4.** **pH dependence of the mGarnet chromophore.** (a) The normalized absorbance at 598 nm, which represents the fraction of anionic chromophores, is plotted as a function of pH (black symbols). The data were fitted by a Henderson-Hasselbalch relation,

$C^{\_}=\frac{1}{1+{10}^{n({pK}_{a}-\mathrm{pH})}},$

with p*K_a_* = 7.4 and *n* = 1.0 (red line). (**b**) Normalized emission intensity at 700 nm (black symbols), plotted as a function of pH. We note that such fluorescence emission data, although oftentimes used to measure such protonation equilibria, do not represent the fraction of anionic chromophores because of the pH dependence of the quantum yield. Here, the emission intensity peaks at pH 8.0 and decreases toward higher pH values. As shown in panel a, however, the population of anionic chromophores further increases above pH 8; the decrease of the emission must, therefore, result from a pH dependence of the quantum yield. A Henderson-Hasselbalch fit yields a lower protein affinity, p*K*_a_ = 6.8, and the steepness of the transition results in *n* = 1.7 (green line). The apparent cooperativity reflects distortions of the curve due to the pH dependent quantum yield. For reference, we have also included a Henderson-Hasselbalch curve describing a protonation reaction with p*K*_a_ = 6.8 and *n* = 1.0 (red line).


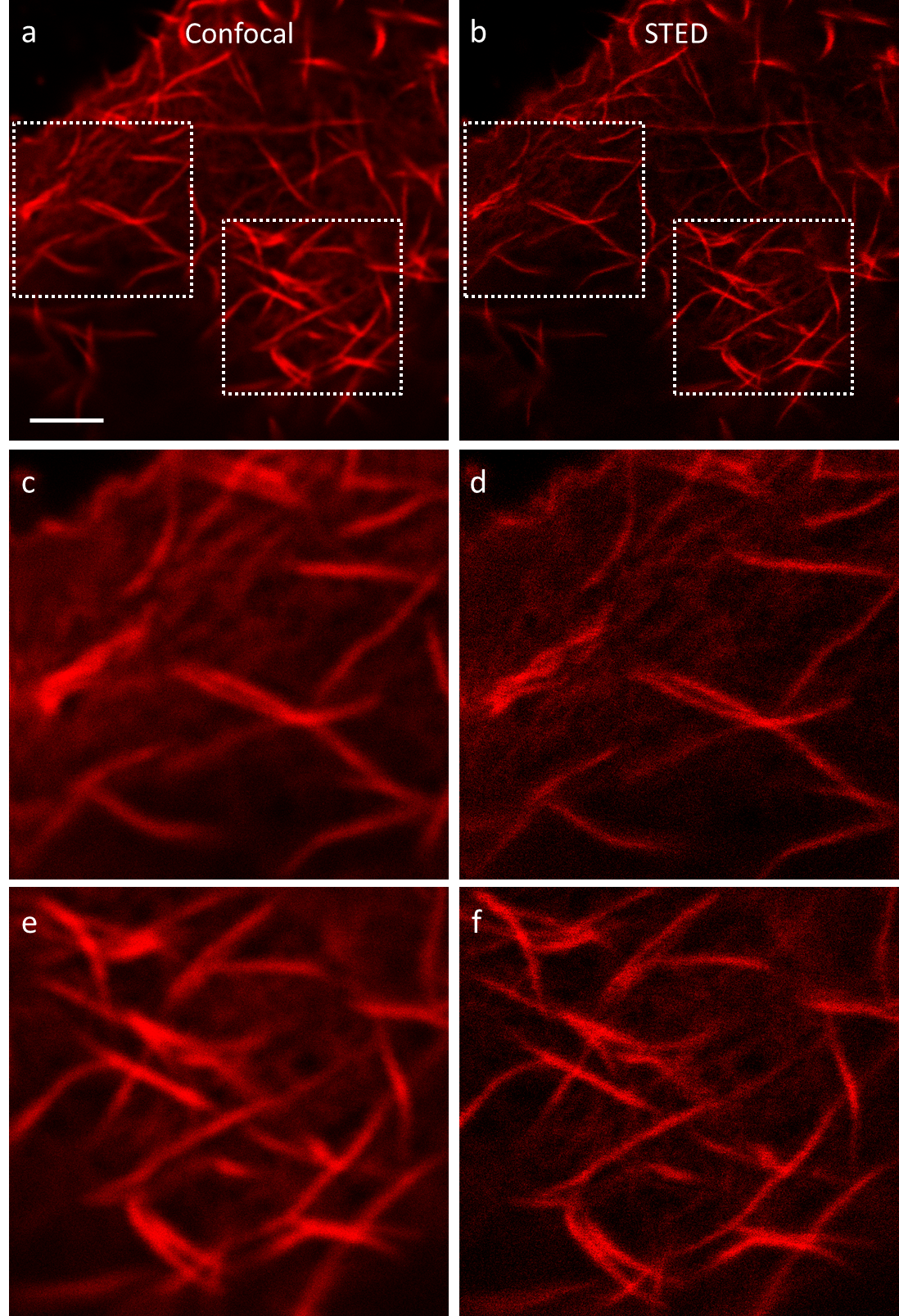


**Supplementary Fig. S5. Fluorescence images of live COS-7 cells transfected with α-actinin-mGarnet.** (a) Confocal and (b) STED image. (c – f) Close-ups of the areas marked by the white frames in (a) and (b). In the STED images (d, f), filaments close to each other are well resolved, which is not the case in the confocal images (c, e). Scale bar, 5 μm.


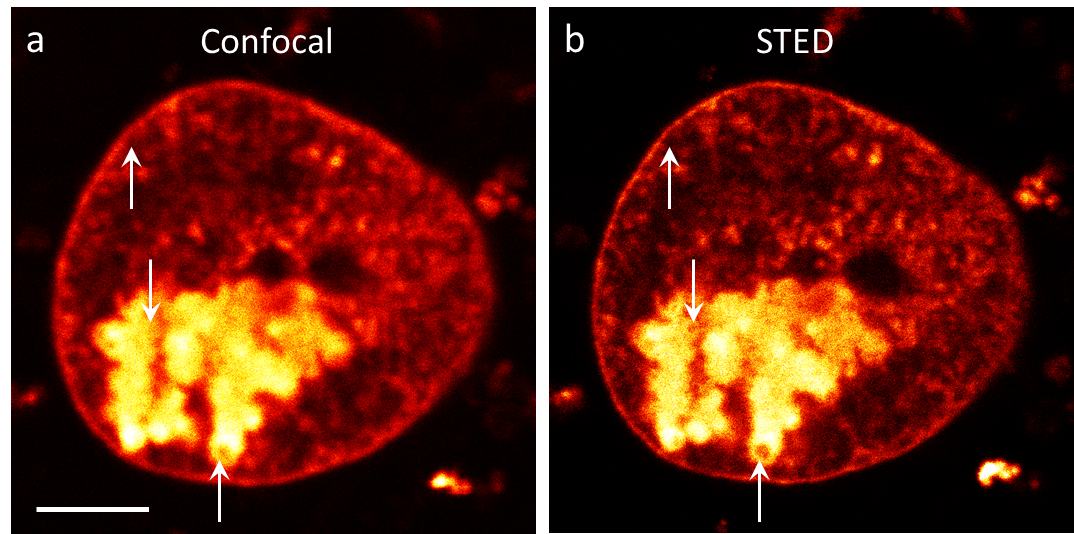


**Supplementary Fig. S6. Fluorescence images of a cell nucleus labeled with H2B-mGarnet.** (a) Confocal and (b) STED image of a live COS-7 cell transfected with H2B-mGarnet, which is visible throughout the nucleus, but with a higher density in DNA-rich regions. The contrast between the observed chromatin structure and the nuclear membrane is significantly improved by using STED, as indicated by the white arrows. Scale bar, 5 μm.


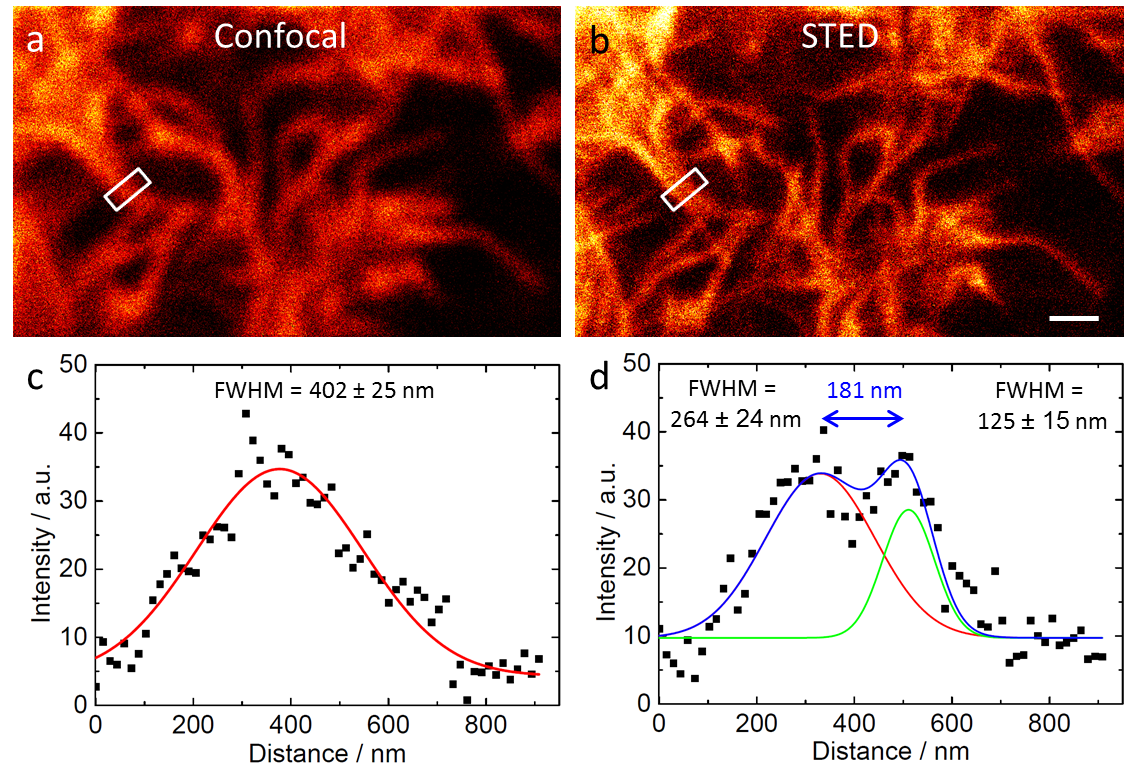


**Supplementary Fig. S7. Confocal and STED images of actin cytoskeletal structures.** (a) Confocal and (b) STED image of a live COS-7 cell transfected with LifeAct-mGarnet (pixel size 14.6 nm). (c, d) A cross-section of the same region indicated by the white boxes in panels (a) and (b) was fitted with Gaussian functions (solid lines) to determine full widths at half-maximum. In the STED image, two filaments with FWHM of 264 nm and 125 nm and a distance of 181 nm can be revealed. Scale bar, 1 μm.


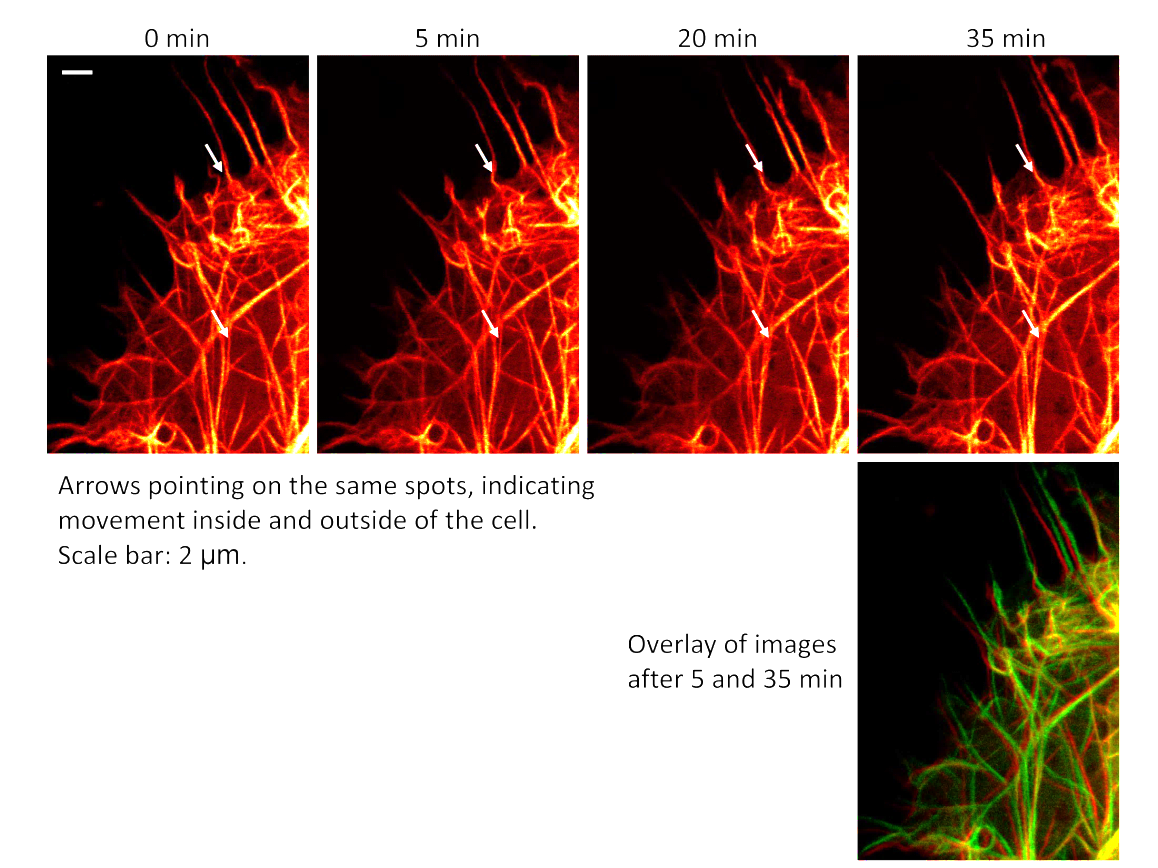

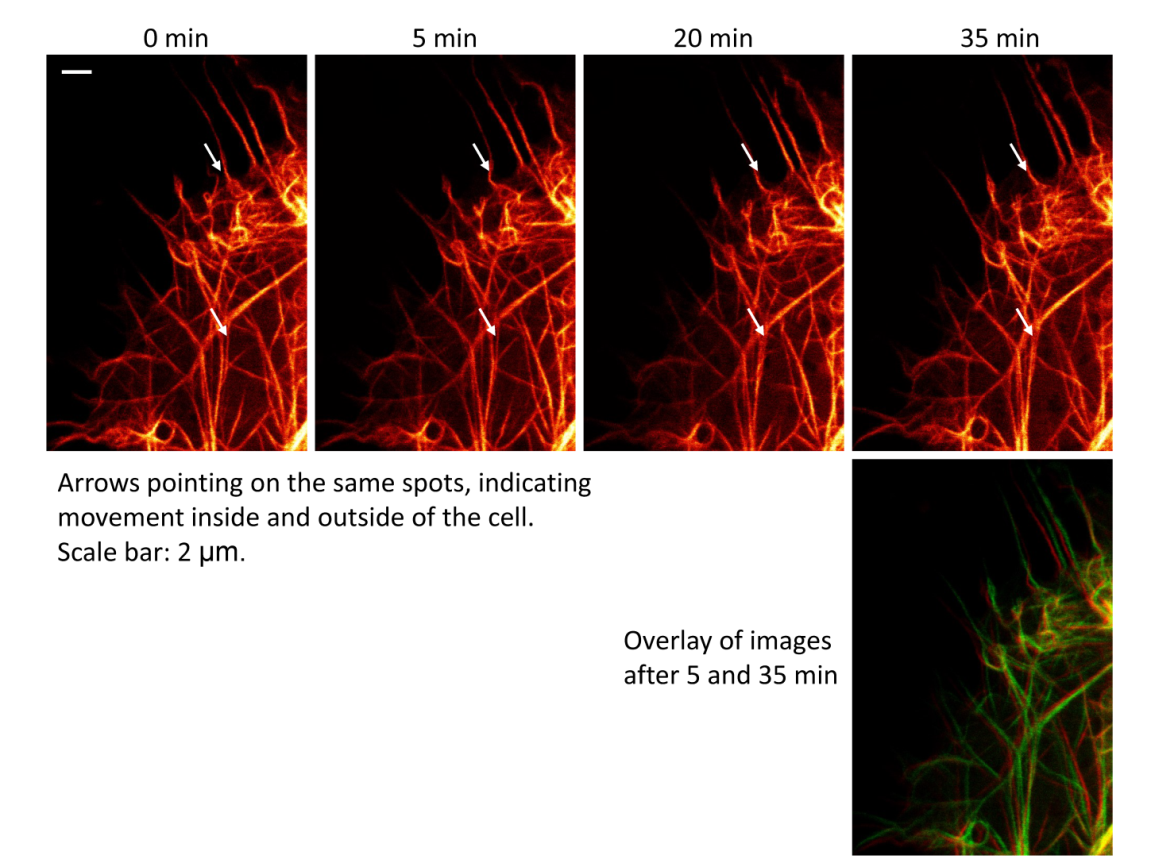


image 1 image 2 image 6 image 10 overlay (#2 / #10)

**Supplementary Fig. S8. STED images of a live COS-7 cell transfected with LifeAct-mGarnet.** We recorded ten images (18 × 28 µm^2^, depletion at 780 nm, laser power 56 mW, dwell time 40 μs, pixel size 20 nm) separated by time intervals of 3 min; images 1, 2, 6 and 10 are displayed. The actin cytoskeletal structure is highly dynamic, as can be inferred from the positions marked by the arrows. The movements are clearly visible in the overlay of image 2 (red) and image 10 (green). Scale bar, 2 µm.


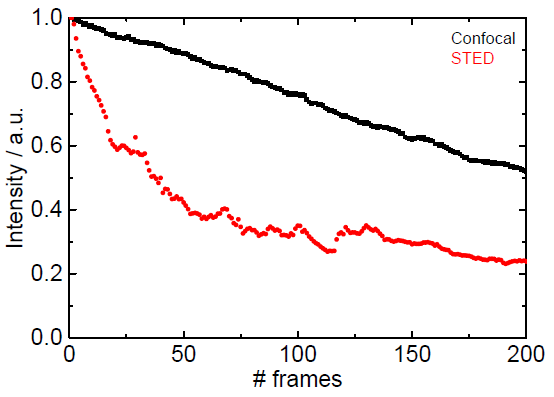

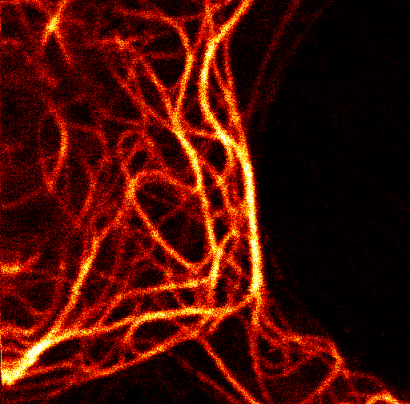

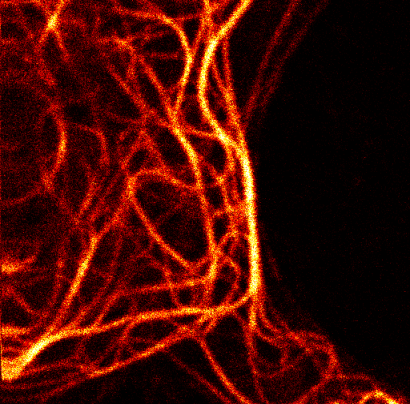

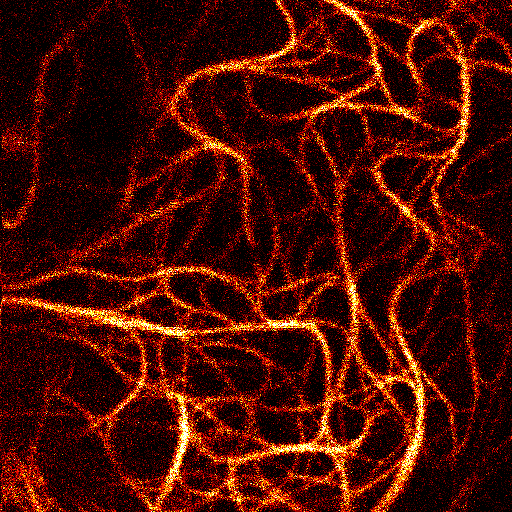

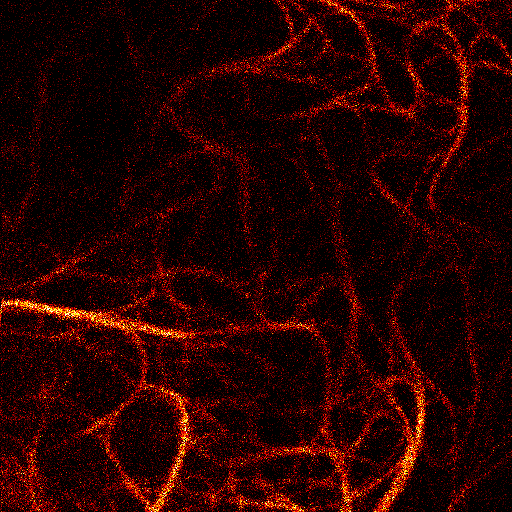


e

a

c

d

b

**Supplementary Fig. S9. Repeated confocal and STED measurements on COS-7 cells labeled with mGarnet-RITA.** Overall, we recorded 200 successive images to analyze photobleaching of mGarnet. (a) Integrated emission intensity as a function of image number. In the confocal setup, there was hardly any loss in emission intensity within the first 40 images ((b) image #1, (c) image #40). In the STED setup, the emission intensity decreased to ~50% between (d) the first and (e) the 40^th^ image. Scale bar, 2 μm. (512 × 512 pixels, pixel dwell time 40 μs, pixel size 40 nm) with excitation at 640 nm (laser power 6.3 μW) and depletion at 780 nm (laser power 46 mW).

**References**

1. Akerboom, J. *et al.* Genetically encoded calcium indicators for multi-color neural activity imaging and combination with optogenetics *Front. Mol. Neurosci.* **6**, 2 (29 pages) (2013).

2. Shcherbo, D. *et al.* Near-infrared fluorescent proteins *Nat. Methods* **7**, 827-829 (2010).
